# Exon definition as a potential negative force against intron losses in evolution

**DOI:** 10.1186/1745-6150-3-46

**Published:** 2008-11-13

**Authors:** Deng-Ke Niu

**Affiliations:** 1 Ministry of Education Key Laboratory for Biodiversity Science and Ecological Engineering, College of Life Sciences, Beijing Normal University, Beijing 100875, PR China

## Abstract

**Background:**

Previous studies have indicated that the wide variation in intron density (the number of introns per gene) among different eukaryotes largely reflects varying degrees of intron loss during evolution. The most popular model, which suggests that organisms lose introns through a mechanism in which reverse-transcribed cDNA recombines with the genomic DNA, concerns only one mutational force.

**Hypothesis:**

Using exons as the units of splicing-site recognition, exon definition constrains the length of exons. An intron-loss event results in fusion of flanking exons and thus a larger exon. The large size of the newborn exon may cause splicing errors, i.e., exon skipping, if the splicing of pre-mRNAs is initiated by exon definition. By contrast, if the splicing of pre-mRNAs is initiated by intron definition, intron loss does not matter. Exon definition may thus be a selective force against intron loss. An organism with a high frequency of exon definition is expected to experience a low rate of intron loss throughout evolution and have a high density of spliceosomal introns.

**Conclusion:**

The majority of spliceosomal introns in vertebrates may be maintained during evolution not because of potential functions, but because of their splicing mechanism (i.e., exon definition). Further research is required to determine whether exon definition is a negative force in maintaining the high intron density of vertebrates.

**Reviewers:**

This article was reviewed by Dr. Scott W. Roy (nominated by Dr. John Logsdon), Dr. Eugene V. Koonin, and Dr. Igor B. Rogozin (nominated by Dr. Mikhail Gelfand). For the full reviews, please go to the Reviewers' comments section.

## Background

### A brief introduction on intron loss during evolution

Spliceosomal introns are sequences that interrupt nuclear genes and will be removed from the corresponding RNA transcripts by spliceosomes. They are found in all eukaryotic groups, but their density, i.e., the average number of introns per gene, varies dramatically among eukaryotes from 8.4 in humans to 0.0075 in the microsporidian *Encepalitozoon cuniculi *[1-4]. While the origin of spliceosomal introns remains under debate [4-7], accumulating evidence suggests that early eukaryotic ancestors had high intron density (perhaps up to several introns per gene) and that the diversity of intron density among eukaryotic genomes largely reflects varying degrees of intron loss [7-14]. However, the mechanism of the differential losses of introns across eukaryotes has not been thoroughly explored. A popular model for intron loss is homologous recombination between intronless cDNA and the corresponding genomic DNA [1,15-18]. In this model, reverse transcription proceeds from the 3' end to the 5' end of template mRNA and often terminates prematurely. A high frequency of cDNA fragments reverse-transcribed from the 3' end of corresponding mRNAs results in the preferential loss of introns at the 3' side of genes via recombination. This model is supported by observations that the introns in intron-poor genomes are biased toward the 5' end of genes [1,2,15,19]. It is generally assumed that the reverse transcriptases are provided by retrotransposons. Organisms with a low level of retrotransposon activity are expected to have low rates of intron loss in evolution [20]. *Plasmodium *species, which lack known transposable elements, have much lower rates of intron loss as compared with other eukaryotic lineages, such as plants, fungi, and nematodes [21]. However, the rates of intron loss in *Plasmodium *species are comparable with those in vertebrates [21], the genomes of which are abundant in retrotransposons [22,23]. Another hypothesis is that segregation of germ and soma during development makes germ line cells express a lower proportion of genes than somatic cells in lower eukaryotes; genes that are expressed only in somatic cells are thus expected to be immune from mRNA-mediated intron losses [24]. By analyzing the whole-genome sequence alignments of human, mouse, rat, and dog, Coulombe-Huntington and Majewski [25] found that most of the intron-loss events occurred within housekeeping genes. However, housekeeping genes still have more introns than tissue-specific genes [26,27]. It seems that the hypothesis involving mRNA-mediated intron loss does not provide a sound explanation of the high density of introns in vertebrates.

The second model of intron loss is genomic deletion via unequal recombination between two copies of genomic DNA [28-30]. To my knowledge, this model provides little insight for the varying degrees of intron losses among eukaryotic genomes. In this study, I propose that the majority of spliceosomal introns in vertebrate genomes may be maintained by a selective force against intron loss: exon definition.

### Exon definition places a length constraint on internal exons

Although most of the key components of spliceosomes are present in all eukaryotic organisms [31], there are two mechanisms of splicing-site recognition: exon definition and intron definition [32-35]. In exon definition, splicing machinery first searches for a pair of splicing sites in every exon. By contrast, in intron definition, the intron itself acts as the unit of recognition, and splicing machinery directly searches for two intronic splicing sites. The splicing machinery of exon definition imposes a length constraint on exons but does not affect intron size. By contrast, the splicing machinery of intron definition limits the size of introns, but not that of exons. Previous studies have shown that exon definition is the major splicing-site-recognition mechanism for vertebrate genes that consist of small exons separated by large introns, while intron definition is common in lower eukaryotes, where small introns are flanked by large exons [32-37]. *In vitro*, expanding the small internal exons of vertebrate genes to 300 nt inhibits the assembly of spliceosomes [38]. Similarly, *in vivo *analysis of a gene with long introns showed that exon size turned out to be problematic when it was expanded to 432 nt, and exon skipping became predominant when the exon was expanded to 526 nt [39].

## Presentation of the hypothesis

**Figure 1:**
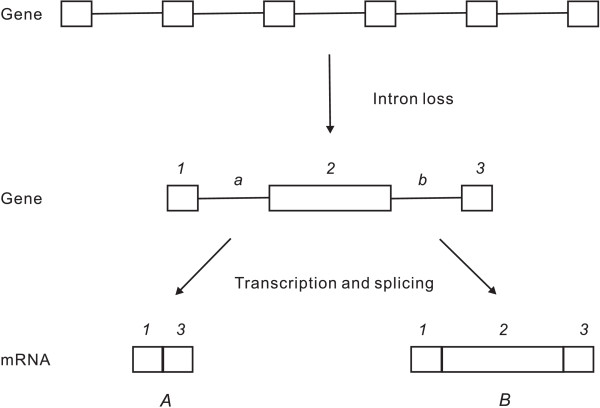
**Intron loss and two different splicing products. **Squares represent exons, and lines represent introns.

Loss of an intron from a gene results in fusion of two flanking exons. Multi-species comparisons of exon-intron structures have shown that adjacent introns tend to be lost in concert [16,20,40] and yield abnormally large exons. Because experimentally expanding exon size results in intron splicing failure or errors [38,39], it is logical to propose that intron loss mutations may affect the splicing of retained adjacent introns if their splicing sites are recognized by exon definition. As shown in Fig. 1, three internal introns are lost from a gene with five introns; four previous exons merge into a large one. From previous studies on intron definition and exon definition [32-39], four patterns can be predicted.

1. In an organism in which exon definition is predominant and introns are very large, intron loss produces a large exon (> 526 nt), which causes exon skipping because exon *2 *is improperly recognized, and splicing fails (Fig. 1). Only truncated mRNA products (transcript *A*) are synthesized. Abnormal proteins will be produced if the mRNA molecules are not degraded by mRNA surveillance mechanisms. Thus, we are inclined to suggest that intron loss notably affects fitness and is under strong negative selection.

2. In an organism in which exon definition is predominant and introns are very large, intron loss produces a middle-sized exon (432–526 nt); both truncated mRNA products (transcript *A*) and normal mRNA products (transcript *B*) will be synthesized. Intron loss partially affects fitness and thus is under weak negative selection.

3. Accumulating evidence indicates that both exon definition and intron definition are effective splicing-site-recognition mechanisms in many eukaryotes [3,39,41,42], although exon definition is prevalent in multicellular animals, whereas intron definition is common in lower eukaryotes [3]. If the remnant introns (introns *a *and *b*) that flank the newly formed large exon (exon *2*) happen to be small, the splicing sites of intron *a *as well as *b *will be recognized by means of intron definition [37,39] and normal mRNA product (transcript *B*) will therefore be synthesized. Loss of those three internal introns will not affect the splicing of nearby introns. Thus, intron loss does not reduce fitness.

4. In lower eukaryotes, in which intron definition is predominant, large exon size does not affect splicing; normal mRNA molecules (transcript *B*) will be produced.

In summary, this hypothesis proposes that the fate of intron loss depends on several factors: splicing-site-recognition mechanisms, the size of the fused exon, and the size of the flanking introns. Certainly, this is a simplified model. Spliceosomal introns themselves may have various functions; for example, they may act as signals for nonsense-mediated decay, harbor regulatory elements, or increase gene expression efficiency [4,43-52]. All of these functions may act as selective forces against intron loss during evolution. On the other hand, positive selection for losing introns is also possible because transcription and splicing of introns cost time and energy [53-55] (but see [56]).

## Testing the hypothesis

According to this hypothesis, the rate of intron loss in evolution depends on the frequency of exon definition and intron definition in a specific organism. Generally, vertebrates that mainly use exon definition have overall lower rates of intron loss than plant and fungi, which mainly use intron definition [25,57]. Significant differences in the rate of intron loss were observed between closely related species, such as *Fugu rubripes *and *Tetraodon nigroviridis *[57]. It will be helpful to study whether corresponding differences exists in the frequency of exon definition and intron definition between these species.

Although vertebrate genes mainly use exon definition, there are still a number of splicing events initiated by intron definition as shown by intron retentions in humans [58]. The occasional intron-loss events throughout vertebrate evolution may happen to flank intron-defined splicing, so the length of remnant flanking introns (introns *a *and *b *in Fig. 1) is not beyond the length limit of intron definition [32,39]. According to our hypothesis, the occasional intron-loss events in vertebrate evolution may also fall into the following categories: 1) the length of the resulting large exon happens to be within the length limit of exon definition [32,39]; 2) the resulting large exon turns into the first or last exon, for which the splicing-site-recognition mechanisms are different from those of internal exons and do not impose size limitations on exons; 3) genes that have lost introns are not essential, and thus the negative selection against intron loss was not efficient; or 4) the intron-loss event occurred in only one copy of a recently duplicated gene, so selection for effective function of the coded protein is relaxed [59]. All of the predictions above are testable.

## Discussion and conclusion

Contrary to what is expected by this hypothesis, the *Plasmodium *species generally splice their introns via intron definition and have low rates of intron loss throughout evolution [20,21]. Besides selective forces, mutation rate and population size also affect the fixation of a mutation. Even if we limit our examination to a selective force, there are many possibilities that affect the rate of intron loss (See "Presentation of the hypothesis"). We could not hope that this simple hypothesis would explain everything in the complex pattern of intron loss in evolution [60]. As suggested by Dr. Koonin, this hypothesis may be applied primarily to chordates (See Reviewers' comments).

If this hypothesis is proved to be true, spliceosomal introns could be maintained in eukaryotes where exon definition is predominant even if they have not any specific function. Spliceosomal introns may recruit adventitious regulatory elements for gene expression; but the recruitments are not necessary for their maintenance in evolution [61]. Further evidence is required to check whether exon definition is a dominant force for maintaining the high intron density of some eukaryotic lineages in evolution. It could be only one of many potential forces that shape the evolution of introns in eukaryotic genomes or even a consequence of the high intron density in some lineages.

## Competing interests

The author declares that he has no competing interests.

## Reviewers' comments

### Reviewer 1: Scott W. Roy, National Center for Biotechnology Information, National Library of Medicine, National Institutes of Health, Bethesda, USA (nominated by John Logsdon)

In this paper, Dr. Niu continues his history of welcome outside-the-box thinking on intron evolution. He points out that intron loss may be more disruptive to splicing of transcripts in the context of exon definition than with intron definition. Consistent with a relationship between exon/intron definition and intron loss rates, he reports that among characterized species, those with more exon definition tend to have more exons overall.

I am unconvinced by this argument. The comparison here is largely between vertebrates – the only lineage known to extensively utilize exon definition – and everything else. Insofar as this is true, this result only restates something that is already known – that vertebrates are unlike most sampled eukaryotes both in their very high intron density and utilization of exon definition. Given the large number of other vertebrate genomic and organismal characteristics that differ from many eukaryotic lineages – large genomes and introns, high content of transposable elements, high degree of alternative splicing, large body size, multicellularity, small population size, etc. – all of which can (and have) been argued to make a difference in both of the traits compared by Dr. Niu, I am unconvinced that this is good evidence for a causal relationship. In addition, since (apparently) simultaneous loss of adjacent introns creates much longer exons than single intron losses, in the context of Dr. Niu's argument it is surprising that such multiple losses seem to account for a relatively large fraction of overall intron losses in many lineages in which such losses are rare (Stajich and Dietrich's work in *Cryptococcs *is the clearest example, but multiple losses are also seen in *Plasmodium *and vertebrates). Finally, it seems very unlikely to me that arguments relying on vague selective differences that are expected to vary considerably across exons within a genome (for instance, the argued effects will presumably be smaller for short exons?) could explain the nearly complete lack of intron loss in, for instance, mammals or *Theileria*. That is, whereas I think Dr. Niu's argument could indeed explain a fewfold reduction in intron loss, I cannot see how it could completely stem intron loss (and gain, for that matter) as seen in some lineages.

#### Author response

*As a hypothesis, this paper just presents a possibility. On the basis of the helpful comments from Dr. Roy and other reviewers (see below), I have revised the manuscript and clarified that this hypothesis is applied primarily to chordates*.

Dr. Niu highlights yet another chicken-and-egg relationship between characteristics of vertebrate genomes: high intron density is likely to favor emergence of exon definition, but the reverse may also be true. Dr. Niu adds another strand to the complex web of cause and effect in genome evolution, and his hypothesis is worthy of consideration for future research.

#### Author response

*I agree with this comment*.

### Reviewer 2: Eugene V. Koonin, National Center for Biotechnology Information, National Library of Medicine, National Institutes of Health, Bethesda, USA

This is an elegant hypothesis that strives to explain, with one unifying idea, two rather puzzling observations: i) the extremely low rate of intron loss in the course of evolution of some groups of animals, particularly, vertebrates, and ii) the typical small size and narrow size distribution of exons in those same organisms. Of course, the two trends are likely to be related as introns loss results in fusion of exons and the formation of a new, long exon, so if intron loss rate is low, there will be fewer long exons. However, the connection needs not be deterministic because exons could expand by other mechanisms as well. Dend-Ke Niu's hypothesis is that exon expansion, by any mechanism, be intron loss, duplication or insertion, is deleterious because splicing in the respective organisms occurs, mostly, via the exon definition.

I find this to be an interesting, credible hypothesis with considerable explanatory power. This being said, there are several issues that deserve some criticism or at least awareness. First, I would suggest that the paper should make it clearer that the hypothesis applies, primarily, to chordates, as intron loss is pretty extensive in other animals like nematodes or arthropods. This does not really detract from the hypothesis but there is no need to claim more generality than justified.

#### Author response

*I agree with this comment and have revised the manuscript accordingly*. 

Second, it should be noticed that that test of the hypothesis illustrated in Figure 2 is weak, and indeed, rather ambiguous because the ratio of the   numbers of exon skipping to intron retention events is a very indirect proxy of exon definition, and intron density is not intron loss either. With regard to the latter, I think that it is possible to use estimates of actual intron loss rates from: Carmel et al. Genome Res. 2007 Jul; 17(7):1034-44, to at least diminish the uncertainty associated with the use of intron density. This is, in a sense, not a crucial issue because the paper is a   hypothesis, and the decisive test in any case remains to be done.

#### Author response

*I thank Dr. Koonin for pointing out this flaw. The related text and figure have been removed from the main article. However, to aid readers' understanding of the reviewing process for this paper, I included them as an online supplementary file (see *Additional File 1).

Third, and perhaps, somewhat more disturbingly, I find the logic in the Discussion regarding exon definition being the ancestral mode of splicing to be weak, at best, and rather hard to follow. First, there is no need to postulate "forces" preventing intron loss at any stage of evolution. Yes, the fact that there was, to our knowledge, virtually, no loss throughout chordate evolution does require a very specific explanation but this does not necessarily apply to the ancestor of multicellular animals. Weak purifying selection owing to a small effective population size would suffice. Accordingly, I do not think there is any reason to believe that exon definition is ancestral. On the contrary, I would maintain that intron definition is the ancestral modality whereas exon definition came to be of major importance in lineages where a massive increase in intron size became possible, e.g., as the result of expansion of mobile elements. Of course, chordates present the prime example. Once many introns became large, the "invention" of exon definition became the only way for these organisms to survive.

#### Author response

*I agree with this comment. The paragraph has been moved to an online supplementary file (see *Additional File 1*) to aid readers' understanding of the reviewing process for this paper*.

I would like to emphasize that the above criticisms do not invalidate Dend-Ke Niu's hypothesis which, on the whole, I find interesting, stimulating, and plausible.

### Reviewer 3: Igor B. Rogozin, National Center for Biotechnology Information, National Library of Medicine, National Institutes of Health, Bethesda (nominated by Mikhail Gelfand)

The author suggested a hypothesis that exon definition is a potential negative force against intron losses. This is an interesting hypothesis, however I have certain reservations regarding this paper.

1) *N. vectensis *and *Arabidopsis *are among the most intron-rich species that contain a lot of ancient introns. However, splicing in *N. vectensis *is largely consistent with the exon definition (the fraction of exon skipping [CE fraction] = 0.87; I hope that I understood correctly this variable suggested by McGuire et al., 2008) whereas splicing in *Arabidopsis *is more consistent with the intron definition (CE fraction = 0.17). This result immediately suggests that the hypothesis is not able to explain the extreme cases of high intron density.

2) Correlation coefficient is a measure of the linear dependence between two random variables. It is obvious that these variables are not random. Many species (e.g. fungi, land plants or deuterostomes) are closely related. Thus the correlation coefficient might simply reflect the non-randomness of the analyzed variables. Removal of closely related species may substantially change results of the correlation analysis (taking into account a huge variance of the CE fraction [see Table 3 in McGuire et al., 2008] and decrease in the size of the dataset).

3) Even if the correlation is significant, this does not mean that there is a true connection between two variables. Pearson correlation coefficient (0.49) is not exciting. There might be some other variables (e.g., some properties of different EST libraries used by McGuire et al., 2008) which would produce a stronger correlation and explain larger fraction of variation in the data.

#### Author response

*I agree with these comments and thank Dr. Rogozin for pointing out these flaws. The relevant text and figure have been deleted from the main text and are now included as an online supplementary file (see *Additional File 1*) to aid readers' understanding of the reviewing process for this paper*.

## Supplementary Material

Additional file 1 **Text and figure removed from the main text according to reviewers' comments. **Presentation of those contents is intended to aid the readers' understanding of the reviewing process for this paper. Click here for file
