# Supplementary material for: Exon definition as a potential negative force against intron losses in evolution
Source: Biol Direct. 2008 Nov 13;3:46. doi: 10.1186/1745-6150-3-46 (PMC2614967; doi:10.1186/1745-6150-3-46)
Supplement: Additional file 1 — Text and figure removed from the main text according to reviewers' comments. Presentation of those contents is intended to aid the readers' understanding of the reviewing process for this paper. [file 1745-6150-3-46-S1.pdf]

Exon definition as a potential negative force against intron losses in evolution

Deng-Ke Niu

Address: Ministry of Education Key Laboratory for Biodiversity Science and Ecological Engineering, College of Life Sciences, Beijing Normal University, Beijing 100875, China

Email: dkniu@bnu.edu.cn or dengkeniu@hotmail.com

The following text and figure were removed from the main text at the recommendation of reviewers. Presentation of these contents is *meant only to aid readers' understanding of the reviewing process for this paper*.

Originally under the subtitle of "Testing the hypothesis"

To my knowledge, there have not been enough statistical data on the rate of intron loss in evolution.

Here, we can substantiate the hypothesis indirectly. As noted above, the variation of intron density among eukaryotic genomes largely reflects the different levels of intron loss [1-8]; it could be reasonable to substitute intron density for the frequency of intron loss in evolution. Because errors in intron definition tend to cause intron retention and those in exon definition tend to produce exon skipping [9], McGuire *et al*. [10] recently used the ratio of exon skipping to the total number of intron retention and exon skipping to represent the frequency for exon definition. By plotting the ratio versus intron density that McGuire *et al*. [10] calculated, we can see a strong positive correlation (n = 39, Spearman's rho = 0.70, $P = 10^{-6}$, Fig. 2). That is, eukaryotes that preferentially use exon definition retained more introns throughout evolution than those that use mostly intron definition.

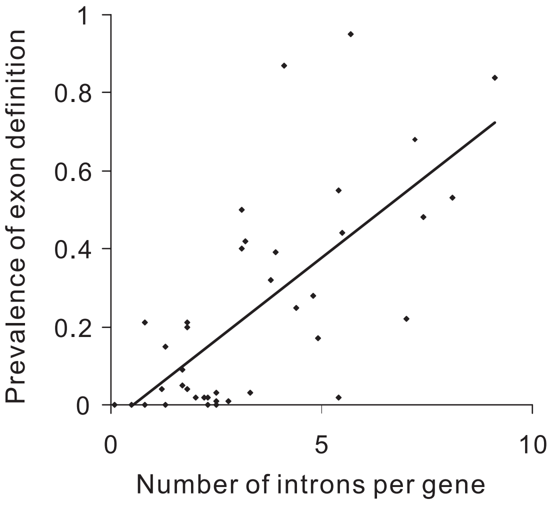

Figure 2 Relationship between the prevalence of exon definition and intron density. For the 39 eukaryotic genomes analyzed in reference [10], the prevalence of exon definition was plotted against intron density. The best-fit line is $y = 0.0845x - 0.0468$ (R^2 = 0.49). Spearman's rank correlation analysis showed that the correlation is highly significant ($P = 10^{-6}$). Data on the number of introns per gene are from the column of intron density in Table 4 of reference [10], and data on the prevalence of exon definition are from the column of "CE fraction" in Table 3 of reference [10].

Originally under the subtitle of "Discussion and conclusion"

The effect of exon definition on the evolution of intron density depends on the scenarios of origin and evolution of exon definition and intron definition. Exon definition is predominant in multicellular animals, whereas intron definition is the principal splicing-site-recognition mechanism in lower eukaryotes [10]. If

intron definition is primordial, and exon definition evolved later [11, 12] and became common in the origin and evolution of multicellular animals, an unknown force must exist to maintain high intron density in the ancestors of multicellular animals. If such a force exists, we are inclined to consider that force being important for the maintenance of high intron density after the origin of multicellular animals, while exon definition acts as a supplemental one. On the other hand, if exon definition was common in very early ancestors and intron definition was derived [13, 14], our hypothesis is more reasonable. In lineages where exon definition became less important, the force that was required to maintain high intron density was released, leading to massive intron losses in evolution. In contrast, in lineages where exon definition continued to be predominant, loss of introns was selected against because of the resulting splicing errors of nearby remnant introns.

1. Rogozin IB, Sverdlov AV, Babenko VN, Koonin EV: **Analysis of evolution of exon-intron structure of eukaryotic genes.** *Brief Bioinform* 2005, **6:**118-134.
2. Roy SW, Gilbert W: **Complex early genes.** *Proc Natl Acad Sci USA* 2005, **102:**1986-1991.
3. Sverdlov AV, Csuros M, Rogozin IB, Koonin EV: **A glimpse of a putative pre-intron phase of eukaryotic evolution.** *Trends Genet* 2007, **23:**105-108.
4. Csuros M, Rogozin IB, Koonin EV: **Extremely intron-rich genes in the alveolate ancestors inferred with a flexible maximum-likelihood approach.** *Mol Biol Evol* 2008, **25:**903-911.
5. Stajich JE, Dietrich FS, Roy SW: **Comparative genomic analysis of fungal genomes reveals intron-rich ancestors.** *Genome Biol* 2007, **8:**R223.
6. Roy SW: **Intron-rich ancestors.** *Trends Genet* 2006, **22:**468-471.
7. Raible F, Tessmar-Raible K, Osoegawa K, Wincker P, Jubin C, Balavoine G, Ferrier D, Benes V, de Jong P, Weissenbach J, et al: **Vertebrate-type intron-rich genes in the marine annelid *Platynereis dumerilii*.** *Science* 2005, **310:**1325-1326.
8. Srivastava M, Begovic E, Chapman J, Putnam NH, Hellsten U, Kawashima T, Kuo A, Mitros T, Salamov A, Carpenter ML, et al: **The *Trichoplax* genome and the nature of placozoans.** *Nature* 2008, **454:**955-960.
9. Berget SM: **Exon recognition in vertebrate splicing.** *J Biol Chem* 1995, **270:**2411-2414.
10. McGuire AM, Pearson MD, Neafsey DE, Galagan JE: **Cross-kingdom patterns of alternative splicing and splice recognition.** *Genome Biol* 2008, **9:**R50.
11. Ast G: **How did alternative splicing evolve?** *Nat Rev Genet* 2004, **5:**773-782.
12. Ram O, Ast G: **SR proteins: a foot on the exon before the transition from intron to exon definition.** *Trends Genet* 2007, **23:**5-7.
13. Collins L, Penny D: **Investigating the intron recognition mechanism in eukaryotes.** *Mol Biol Evol* 2006, **23:**901-910.
14. Kurland CG, Collins LJ, Penny D: **Genomics and the irreducible nature of eukaryote cells.** *Science* 2006, **312:**1011-1014.
